# Supplementary figures and images for: Failure to mate enhances investment in behaviors that may promote mating reward and impairs the ability to cope with stressors via a subpopulation of Neuropeptide F receptor neurons
Source: PLoS Genet. 2024 Jan 18;20(1):e1011054. doi: 10.1371/journal.pgen.1011054 (PMC10795991; doi:10.1371/journal.pgen.1011054)

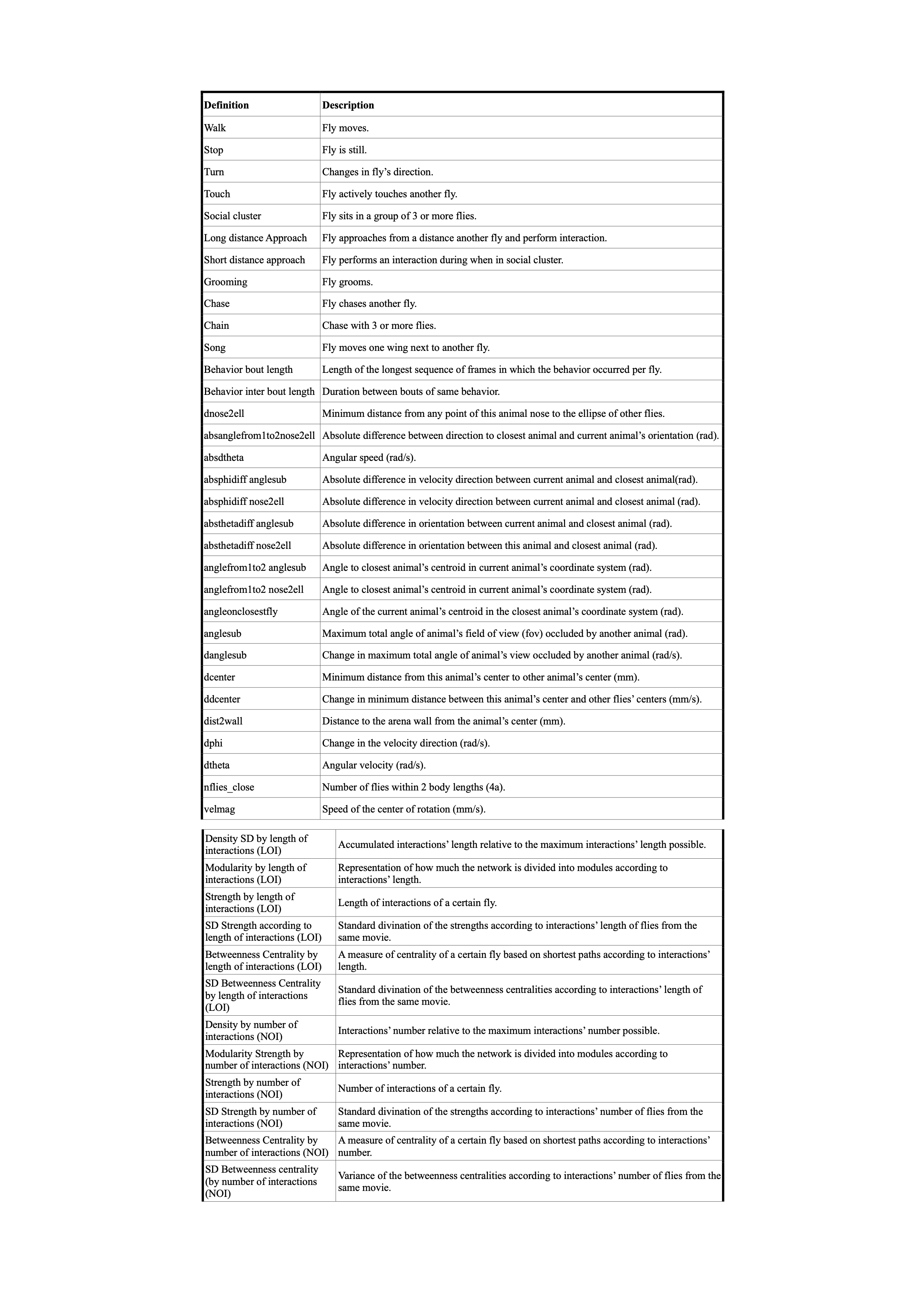

Supplement: S1 Fig — (TIFF) [file pgen.1011054.s001.tiff]

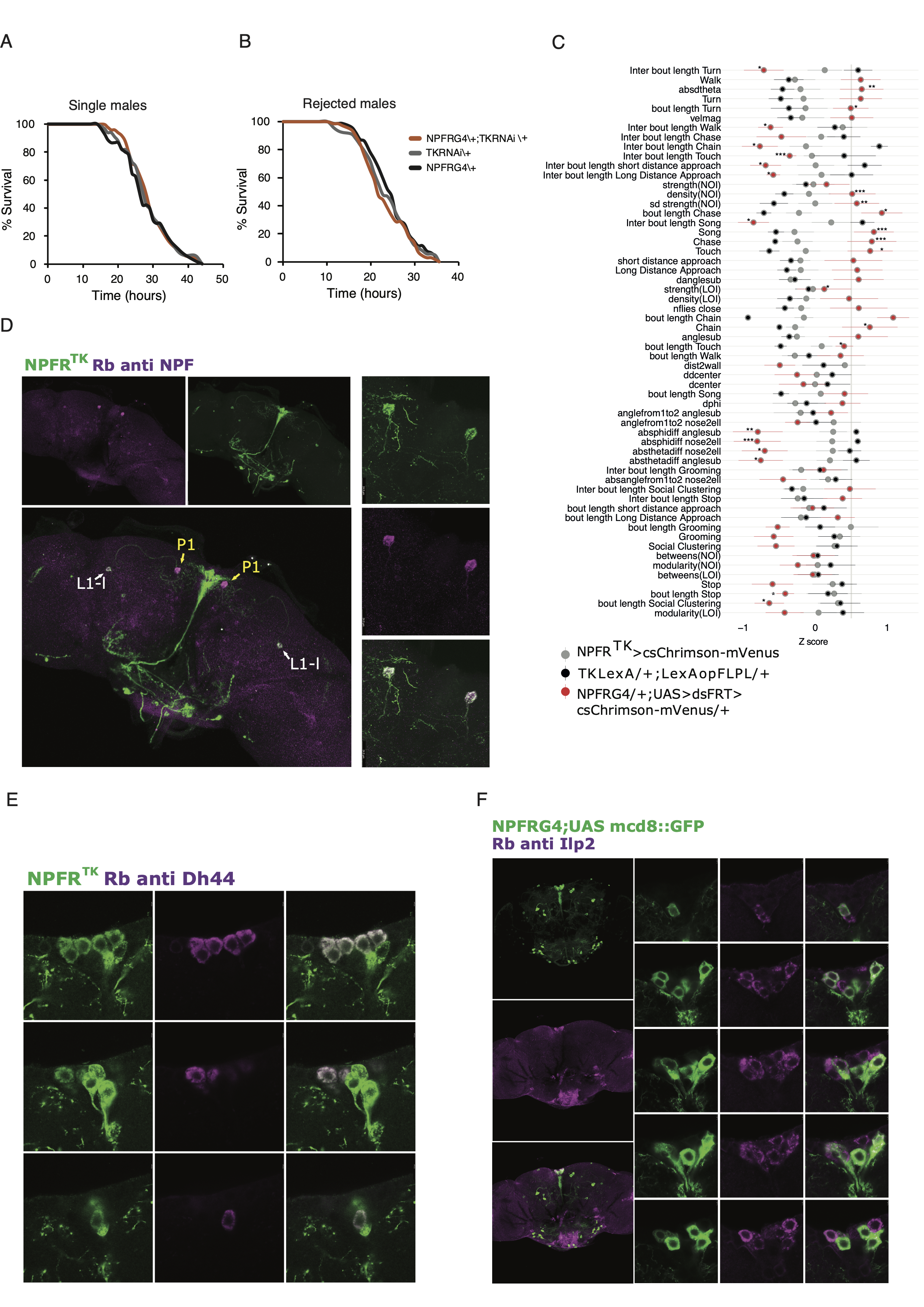

Supplement: S8 Fig — A. Knock down of tk in NPFR neurons does not affect sensitivity to starvation of na.ve-single. Experimental single housed NPFRG4/+;tkRNAi/+ (orange, n = 50) and the genetic controls TK RNAi/+ (gray, n = 33) and NPFR G4/+ (black, n = 45). B. NPFRG4/+; tkRNAi/+ males and their genetic controls were subjected to rejection and their resistance to starvation was assayed. No significant difference in resistance to starvation in NPFR Gal4;tk RNAi flies (orange, n = 79) compared to genetic controls (gray, n = 90 and black, n = 70) was observed. Pairwise log-rank test with FDR correction for multiple comparisons was performed for A,B. C. Behavioral signatures of male-male social interaction within the FlyBowl system during the optogenetic activation of NPFR-TK neurons. n = 13 for NPFRTK(red). n = 13 TK-LexA;LexAop-FLPL (black), and n = 12 NPFRG4;UAScsChrimson-mVenus (gray). *p<0.05, **p<0.01, ***p<0.001. ANOVA or Kruskal-Wallis with post-hoc Tukey’s or Dunn’s test with FDR correction for multiple comparisons was performed. D. Right: colocalization of NPFRTK neurons (green) and NPF+ neurons (magenta, endogenous NPF expression), indicated by arrows. White arrows indicate L1-l neurons, yellow arrows indicate P1 neurons. Left: A closeup to two NPFRTK NPF+ neurons (P1). E. Six NPFRTK (green) neurons colocalize with DH44 (magenta, endogenous Dh44 expression). F. Colocalization of NPFR neurons (NPFR>mCD8-GFP marked in green) with Dilp2 peptide (magenta anti-Dilp2 antibodies). (TIFF) [file pgen.1011054.s008.tiff]
